# JACMP – Founding and 2000–2004

**DOI:** 10.1002/acm2.14431

**Published:** 2024-06-21

**Authors:** Michael Mills

**Affiliations:** ^1^ Radiation Oncology University of Louisville Louisville Kentucky USA

## 1 FOUNDING THE JACMP

In this Section I want to talk about my personal experiences while founding the JACMP, serving as its third and fifth Editor‐in‐Chief and bringing it to profitability (twice!). Those who wish to read a committee and process history of the JACMP's founding may want to read the first Editorial of 2013 (Volume 14, Issue 1).^1^ However, for this chapter, I want to share what I learned in this adventure as a guide in the future for those with similar ambitions and callings.

It was October of 1997, and my colleague Ann Wright was visiting my town on a business consult. We walked on the shore of a nearby lake and began talking about the needs of medical physicists who were not in research positions. Many of us faced patient‐treatment issues and the lack of communication, but there was no medical physics academic clinical journal. Often our colleagues were re‐inventing the wheel. This was a problem from two perspectives; obviously there was much time wasted in the duplication of effort. But also, solutions were realized inconsistently, potentially leading to uneven patient care. As we discussed the possibility of a clinical journal, it was apparent to us that only if the journal leadership always put the patient first would we arrive consistently at the correct destination. We reasoned that what is right for the patient and right for the author is ultimately right for the community of medical physicists and the organization.

Founding a journal is always a complex and risky endeavor. However, we did not know just how challenging this would be in the late 1990s. Even today, many journals lose money for their societies. It was not unusual for a journal to struggle for over 20 years to become profitable. Additionally, print publication and the associated revenue from print ads would slowly become an anachronism not just in radiology or medicine, but throughout the publishing industry.

We faced the dilemma early on about whether to provide the articles freely (open access) or to charge to view and download through a subscription. We were driven back to the principal above—what is best for the patient. We concluded that making articles freely available to the world would provide the greatest benefit to the patient. We did not shrink from this conclusion, but rather embraced it. A committee was formed 1998 and we worked on these concepts for about a year.

In May of 1998, I came to interview for a position at the University of Louisville. It was Derby weekend, and hotel rooms were difficult to come by. When I visited Peter Almond in his office, we had a lot to discuss—he spoke about the position he was vacating and I spoke to him about the need for the JACMP to have an Editor‐in‐Chief. In the end, both of us decided the respective positions made sense.

At that point, I took a step back from making decisions about the new journal. Peter made an announcement to the *Medical Physics* Board of Editors about this new clinical journal at the AAPM Annual Meeting in Nashville in the Summer of 1999. Peter selected the publisher, and the initial cast of Associate Editors. He used his influence to identify authors and topics for the inaugural JACMP issue which was published the second week of February in the year 2000.

## 2 THE FIRST 5 YEARS

When the first JACMP issue was published, there was initially not a lot of publicity or excitement. However, the existence of the new journal began to spread by word of mouth and submissions began to trickle in. Peter no longer had to recruit for authors and articles after the second issue was published.

Best Paper Awards were part of the JACMP culture from the very beginning. Initially sponsored by advertising vendors, The year 2000 Best Paper Awards are listed below.

The 2000 Best Paper awards from the JACMP were as follows:

1. The winner of the “LAP Award for Excellence for the Best Radiation Oncology Physics Paper”: “Guide to clinical use of electronic portal imaging” by Michael G. Herman, Jon J. Kruse, and Christopher R. Hagness.
2. The winner of the “RIT Award for Excellence for the Best Diagnostic Physics Paper”: “Protecting patients by training physicians in fluoroscopic radiation management” by Benjamin R. Archer and Louis K. Wagner.
3. The winner of “The Elekta Award for Excellence for the Best Professional Paper”: “Preventative maintenance and unscheduled downtime from an economic perspective” by Peter Dunscombe, Gisele Roberts, and Lianne Valiquette.

The JACMP was operationally sound and growing, but at the end of 2000 it was apparent that the anticipated growth would soon place demands on support finances. Charging a fee for access was seriously considered, but in the end, the Editors could not envision restricting the benefit of these articles to the worldwide community. In 2001, we were still 2 years away from the worldwide open access revolution and the Bethesda, Berlin, and Budapest Conferences and initiatives. Still, some open access journals in other fields were beginning to have a presence. All such journals faced economic challenges as one would expect when you give the product away for free. This is especially true when a Journal is free to submit, free to publish and free to access.

In 2001, some of the Editors made a serious effort to solicit banner ads and other sources of revenue for the JACMP. I took a lead role in this. However, journal costs increase with the number of articles published. By the end of 2001, we needed another solution. During the year 2001, some new features were added to the JACMP, including Book Reviews and Letters to the Editor. Additionally, the decision was made at the end of 2001 to maintain the open access publication model for the next year and hopefully for the foreseeable future.

During early 2002, JACMP became an official journal of the International Organization for Medical Physics (IOMP). This reflected the growing reality that the JACMP was beginning to enjoy an international presence. In addition to the United States and Canada, papers came from 14 other countries in 2002. During 2002–2003, the AAPM considered publishing the JACMP as a quarterly clinical online supplement, but ultimately this idea was not supported. Finally, Peter Almond decided that 2002 would be his last year as JACMP Editor. We were all grateful for the job he did and the excellence of the clinical content of JACMP published articles. Ed McCullough graciously accepted the open position and was named as JACMP Editor‐in‐Chief for 2003. Subsequently the number of JACMP published articles increased by almost 20% that year.

By 2003, some things in the publishing landscape began to change for the better. The aforementioned Open Access meetings took place in the cities beginning with “B.” Of great significance, the first version of the Public Knowledge Project Open Journal Systems (OJS) software was released to the public and available for use without cost. This software was a complete tool to establish a journal, manage submissions and assignments for peer review, and to approve and publish articles online. The OJS reduced the cost of publishing the JACMP by tens of thousands of dollars per year.^2^ Secondly, the work of the Editor‐in‐Chief would be donated. Thirdly, there was a need to write a Request for Proposal (RFP) for publishing services as we needed to find a new lower cost publishing partner. I learned what was needed to write the RFP which ultimately led to Multimed, of Toronto Canada, being selected as the successor JACMP publisher. Finally, the Journal leadership redoubled its efforts to sell banner ads within this new publication model (many thanks to Ed Nickoloff and Jim Astarita). With these changes, the JACMP could be operated at breakeven or profit if it published 75 articles per year or fewer. Above the 75‐article limit, publishing an article would cost the organization about $500 dollars per article. This bought us a few years until the inevitable growth once again strained the limits of financial support.

For 2004, the JACMP initiated a double‐blind review process—meaning the authors are unaware of the identity of the reviewers and neither do the reviewers have any information respecting the identity of the authors. The double‐blind process is probably the fairest to scholars, as it helps eliminate any bias in favor of well‐known scholars and programs.

Some JACMP statistics for the first 5 years (2000–2004).

**Table:** 

Year	Editor‐in‐Chief	Deputy EIC	Number of issues	Number of articles
2000	Peter Almond		4	24
2001	Peter Almond		4	27
2002	Peter Almond		4	36
2003	Ed McCullough		4	44
2004	Michael Mills	Tim Solberg	4	31

The uncertainty surrounding the JACMP took a toll on the number of papers published in 2004, but this was a temporary setback. In the later part of the decade, the JACMP would once again face the limits of its financial model. By 2016, there was little choice but to move to an author funded Article Publication Charge (APC) in order to stay in business while publishing over 250 articles per year.

I do not want to end this article without mentioning some remarkable people who served as our publishing partners after the transition. The team from Multimed—Jill Torigian, President, the Shand sisters (Laura Hope, Amanda Shand, and Heather Seunath), and the remarkable Ann‐Fothergill Brown—JACMP copy editor, all of whom increased the quality of the JACMP in every respect. Finally, I want to mention an unnamed vendor who guaranteed the journal would break even during the last year under the old financial model. We are very grateful for your sensitivity to our struggle to get the Journal started and for your generosity.

## CONFLICT OF INTEREST STATEMENT

The author declares no conflicts of interest.
